# Common and uncommon neuroimaging manifestations of ataxia: an illustrated guide for the trainee radiologist. Part 2 - neoplastic, congenital, degenerative, and hereditary diseases

**DOI:** 10.1590/0100-3984.2021.0112

**Published:** 2022

**Authors:** Vinicius de Menezes Jarry, Fernanda Veloso Pereira, Mariana Dalaqua, Juliana Ávila Duarte, Marcondes Cavalcanti França Junior, Fabiano Reis

**Affiliations:** 1 Department of Radiology, Universidade Estadual de Campinas (Unicamp), Campinas, SP, Brazil.; 2 Hôpitaux Universitaires de Genève, Service de Radiologie, Geneva, Switzerland.; 3 Department of Radiology and Diagnostic Imaging, Hospital de Clínicas de Porto Alegre (HCPA), Porto Alegre, RS, Brazil.; 4 Department of Neurology, Universidade Estadual de Campinas (Unicamp), Campinas, SP, Brazil.

**Keywords:** Neuroimaging, Cerebellar ataxia, Cerebellar nuclei, Magnetic resonance imaging, Neuroimagem, Ataxia cerebelar, Núcleos cerebelares, Ressonância magnética

## Abstract

Ataxia is defined as a lack of coordination of voluntary movement, caused by a variety of factors. Ataxia can be classified by the age at onset and type (chronic or acute). The causative lesions involve the cerebellum and cerebellar connections. The correct, appropriate use of neuroimaging, particularly magnetic resonance imaging, can make the diagnosis relatively straightforward and facilitate implementation of the appropriate clinical management. The purpose of this pictorial essay is to describe the imaging findings of ataxia, based on cases obtained from the archives of a tertiary care hospital, with a review of the most important findings. We also discuss and review the imaging aspects of neoplastic diseases, malformations, degenerative diseases, and hereditary diseases related to ataxia.

## Abstract (pt)

Ataxia é definida como uma síndrome de falta de coordenação dos músculos de movimentação voluntária. Vários fatores podem causar ataxias, as quais podem ser classificadas de acordo com a idade, tipo de evolução (crônica ou aguda), cujas lesões envolvem o cerebelo e as conexões cerebelares. Com o uso correto e apropriado da neuroimagem, particularmente da ressonância magnética, o diagnóstico pode ser relativamente direito e o manejo clínico pode ser implementado de maneira correta. O objetivo deste artigo é descrever os achados de imagem na síndrome atáxica a partir de casos recuperados do arquivo digital de um hospital terciário, com a revisão dos principais achados de imagem. Neste ensaio revisamos e discutimos os aspectos de imagem de doenças neoplásicas, malformações, doenças degenerativas e doenças hereditárias relacionadas à ataxia.

## INTRODUCTION

Ataxia is defined as a lack of coordination of voluntary muscle movement, caused by a variety of factors. Its manifestations include gait ataxia, dysarthria, nystagmus, sensory and truncal ataxia, dysdiadochokinesia, intention tremor, dysmetria, and eye movement disorders^(1)^. In this pictorial essay, we discuss and review the imaging aspects of neoplastic diseases, malformations, degenerative diseases, and hereditary diseases.

Posterior fossa brain tumors are most common in the pediatric population, being the most common solid tumors in children, accounting for 54-70% of all central nervous system brain tumors in this population^(2)^.

Cerebellar malformations may be now diagnosed in pregnancy and may be classified as predominantly involving the cerebellum or the cerebellum and brainstem together, the latter scenario occurring earlier in the development. Those conditions may be part of broader syndromes^(3)^.

Among the genetic causes of ataxia, the most common pattern of inheritance is the autosomal recessive pattern, which typically first appears before 20 years of age. Other hereditary types include mitochondrial diseases and lysosomal disorders^(3)^. The degenerative causes of ataxia constitute a heterogeneous group of conditions, including hereditary and non-hereditary conditions, that are associated with late-onset ataxia and may be accompanied by other symptoms, such as parkinsonism and dystonia^(4)^.

The aim of this article is to review various possible causes of ataxia, on the basis of magnetic resonance imaging (MRI) studies obtained from the archives of a tertiary care hospital. The main imaging aspects of the conditions discussed in this article are summarized in Table 1.

**Table 1:** The main imaging aspects of ataxia caused by neoplastic, congenital, degenerative, and hereditary diseases.

Disease	Etiology	Imaging findings
Lhermitte-Duclos disease	Neoplastic	Alternating layers of isointensity and hypointensity on T1 weighted image (T1WI); hyperintense on T2WI. No restricted diffusion; usually no enhancement.
Medulloblastoma	Neoplastic	CT: hyperdense posterior fossa masses with contrast enhancement. MRI: isointense to hypointense on T1WI; hypointense to hyperintense on T2WI; restricted diffusion; and variable contrast enhancement. When desmoplastic, usually heterogeneous (with microcysts). There is earlier meningeal involvement. Mandatory investigation of the neuraxis.
Pilocytic astrocytoma	Neoplastic	Cyst-like lesion with an enhancing mural nodule, isointense to hypointense on T1WI and isointense to hyperintense on T2WI. Mandatory investigation of the neuraxis.
Ependymoma	Neoplastic	Heterogeneous lesion, usually in the posterior fossa: hypointense on T1WI; hyperintense on T2WI; intermediate to high intensity on FLAIR, heterogeneous enhancement; restricted diffusion in the solid component; hyperperfusion; and elevated choline/NAA ratio. Investigation of the neuraxis is mandatory.
Dandy-Walker malformation	Congenital	Enlarged posterior fossa with cerebellar vermis malformation, cyst-like appearance of the fourth ventricle, and superior displacement of the venous torcula.
Progressive ataxia and palatal tremor	Degenerative	Cerebellar and brainstem atrophy; hypertrophic olivary hyperintensity on T2WI/FLAIR images possible in the early stages.
Friedreich’s ataxia	Genetic	Cervical spinal cord, pons, cerebellar peduncles, and cerebellar involvement.
Machado-Joseph disease	Genetic	Atrophy of the cerebellum, brainstem, frontal lobe, globus pallidus, and (especially) superior/middle cerebellar peduncles.

CT, computed tomography; FLAIR, fluid-attenuated inversion recovery; NAA, N-acetylaspartate.

## NEOPLASTIC DISEASES

### Lhermitte-Duclos disease

**Figure 1:**
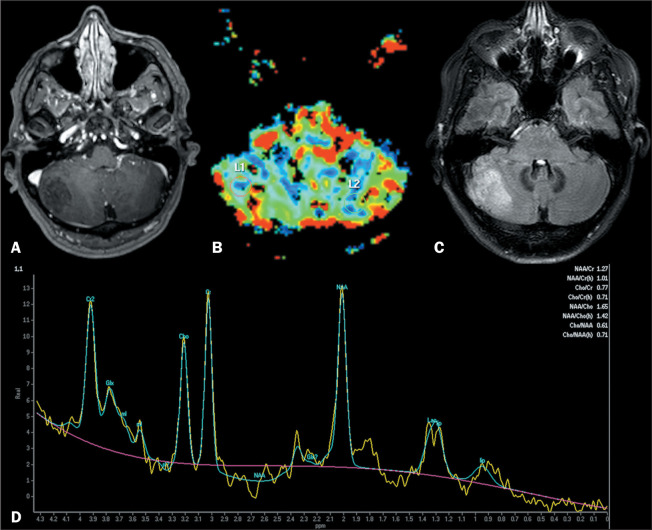
Contrast-enhanced T1WI showing a lesion with a hypointense signal in the right cerebellar hemisphere, featuring alternating layers of isointensity and hypointensity with mass effect (A), with no enhancement or high perfusion (relative cerebral blood volume) on T2* perfusion mapping (B), and a heterogeneous hyperintense signal, with a striated, “corduroy” appearance due to widening of the cerebellar folia in a fluid-attenuated inversion recovery sequence (C). Spectroscopy shows normal metabolic pattern (D). The histopathological diagnosis was Lhermitte-Duclos disease.

Lhermitte-Duclos disease, or dysplastic cerebellar gangliocytoma, is a rare entity^(5-7)^ associated with the phosphatase and tensin homolog, a tumor suppressor gene, the alteration of which results in replacement of the cerebellar internal granule cell layer^(7)^ with loss of normal structure, leading to thickening and enlargement of the cerebellar folia^(6)^. Lhermitte-Duclos disease presents as a unilateral cerebellar lesion with hemispheric expansion, showing parallel linear striations without restricted diffusion and typically no contrast enhancement^(5,6)^, as shown in Figure 1. On perfusion imaging, the relative cerebral blood volume is elevated in most cases. On MR spectroscopy, choline and myoinositol peaks are low, whereas the lactate peak is elevated^(5)^.

### Medulloblastoma

**Figure 2:**
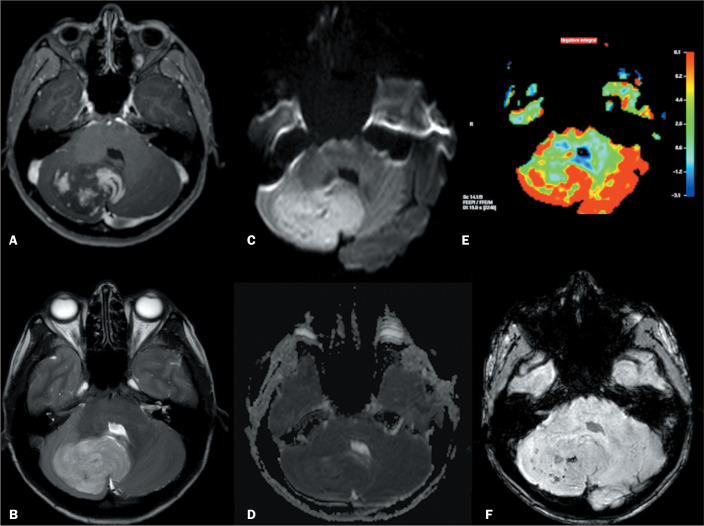
Tumor showing heterogeneous enhancement on gadolinium contrast-enhanced axial T1WI (A), located in the right cerebellar hemisphere and cerebellar vermis, with a hyperintense signal on T2WI (B), restricted diffusion on diffusion-WI (C,D) and high relative cerebral blood volume on T2* perfusion mapping (E), and a focus with a markedly hypointense signal on susceptibility-WI (F). The histopathological diagnosis was desmoplastic medulloblastoma (the molecular classification was not available).

Medulloblastoma is a malignant neuroepithelial mass originating from primitive, undifferentiated cells located in the superior medullary velum^(8,9)^. There are various histological types of medulloblastomas^(10)^: classic; desmoplastic/nodular; extensively nodular; large cell; and anaplastic. They can also be grouped by molecular pattern-the Shh pathway; the Wnt pathway (best prognosis); group 3 (worst prognosis); and group 4-all with different prognoses, anatomical locations, and demographic characteristics^(11)^. Medulloblastomas in the Shh group have two peaks of incidence, one in infancy (< 4 years of age) and another in adulthood (> 16 years of age). They typically give rise to the large-cell, anaplastic, or desmoplastic histological type^(11)^ and are frequently located lateral in cerebellar hemispheres. On computed tomography, classic medulloblastomas appear as hyperattenuating masses, usually located along the midline and with contrast enhancement^(9,10)^. On MRI (Figure 2), they show restricted diffusion and variable enhancement, a pattern that can mimic cerebellar lymphoma^(12,13)^. Intralesional cysts can be found^(8-11)^. MR spectroscopy can depict a high choline peak^(8,10)^ and a taurine peak at 3.4 ppm^(10)^. The desmoplastic type is characterized by atypical features^(8,11)^, such as the location in the cerebellar hemispheres and the more heterogeneous appearance (with microcysts).

### Pilocytic astrocytoma

**Figure 3:**
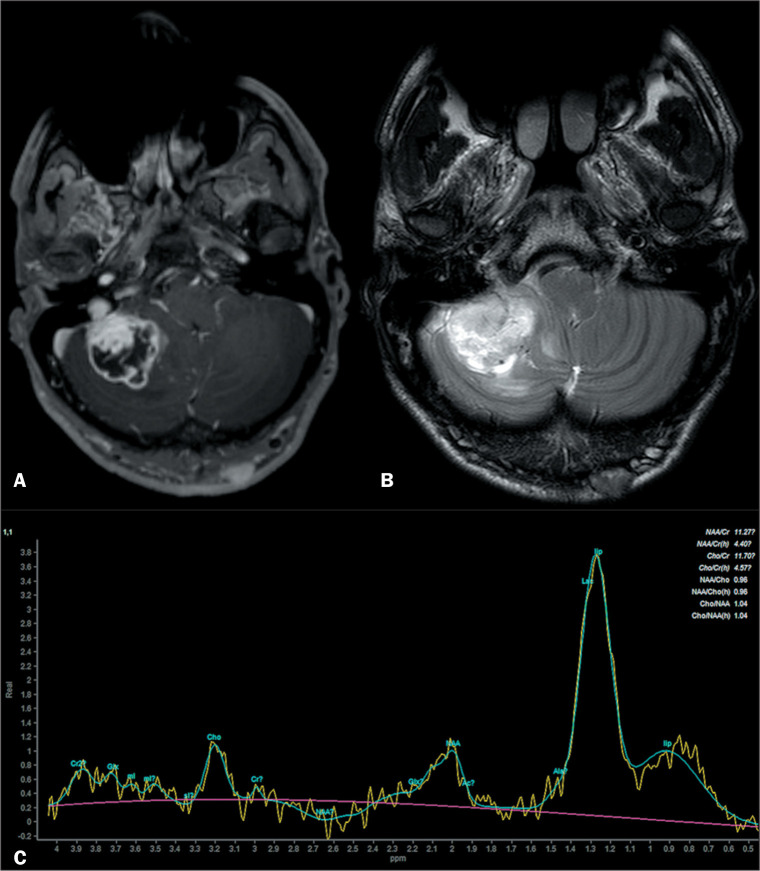
Heterogeneously enhancing lesion on gadolinium contrast-enhanced axial T1WI (A), with a hyperintense signal on T2WI (B). Spectroscopy (C) showing elevation in the choline/creatine ratio (denoting high cellular turnover), as well as in the lipid and lactate peaks. There was no restricted diffusion (not shown). The patient was submitted to a biopsy and diagnosed with pilocytic astrocytoma.

Pilocytic astrocytoma usually presents in the first two decades of life^(14)^ and has been classified as a grade I neoplasm by the World Health Organization^(15)^. On imaging, pilocytic astrocytoma usually presents with one of three patterns^(14,15)^: a large cystic mass lesion with a mural nodule (Figure 3); a mass with a central nonenhancing area; or a predominantly solid mass.

### Ependymoma

**Figure 4:**
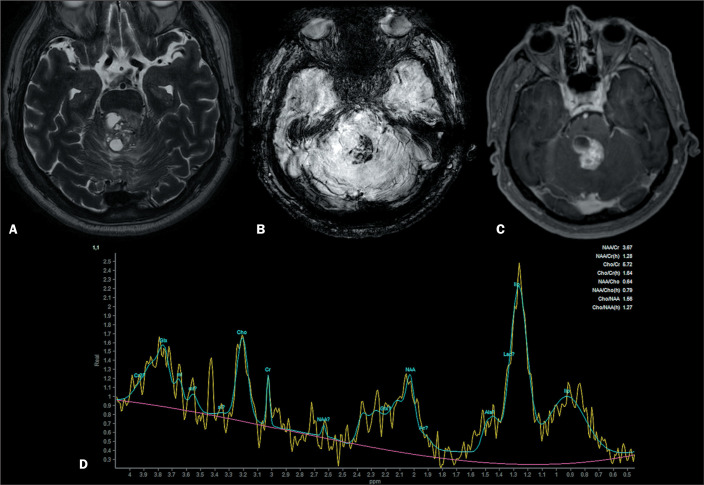
T2WI (A) demonstrating a heterogeneous lesion with cystic areas in the floor of the fourth ventricle, with hypointense components on susceptibility-WI (B) and heterogeneous enhancement on gadolinium contrast-enhanced T1WI (C). On spectroscopy (D), there is an elevated choline peak (high cellular turnover); reductions in the peaks of N-acetylaspartate and creatine; and elevated peaks of lipids and lactate (indicative of necrosis and anaerobiosis, respectively). The final diagnosis was ependymoma.

There are two molecular groups of infratentorial ependymomas: type A and type B. Type A ependymomas occur in very young children and have a poorer prognosis, whereas type B ependymomas occur in older children/adolescents and have good prognosis^(2)^. Imaging can help to distinguish between the two types^(2)^: type A ependymomas usually arise from the lateral recess of the fourth ventricle; and type B ependymomas arise along the midline from the obex. On computed tomography, they appear as heterogeneous masses with contrast enhancement. The MRI findings are demonstrated in Figure 4. They often have calcifications (50%) and, on T2WI, may show hemorrhage foci with very low signal intensity^(16,17)^. Infratentorial ependymomas arise from well differentiated ependymal cells lining the floor of the fourth ventricle and have a “plastic behavior”, passing through the Magendie and Luschka foramina^(16,17)^.

## CONGENITAL DISEASES

### Dandy-Walker malformation

**Figure 5:**
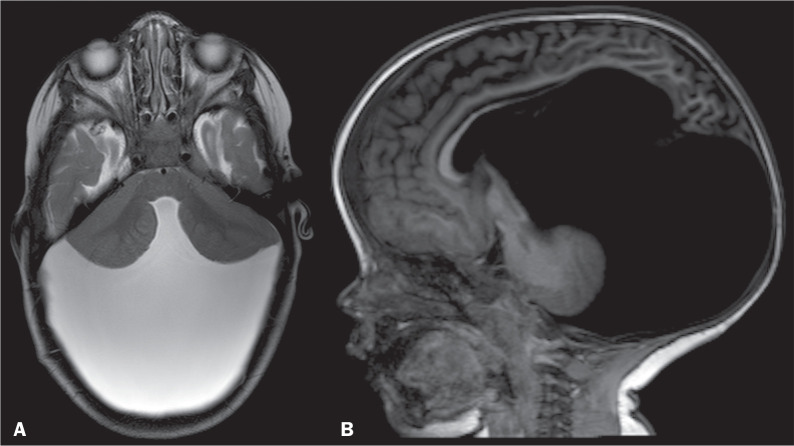
Axial T2WI (A) showing hypogenesis of the cerebellar vermis, with a cyst-like formation. Sagittal T1WI (B) showing agenesis of the posterior portion of the corpus callosum, an enlarged posterior fossa, and an abnormally high tentorium. The patient was diagnosed with Dandy-Walker malformation.

A Dandy-Walker malformation is the most common posterior fossa malformation^(18)^. It may be associated with malformations, including dysgenesis or agenesis of the corpus callosum, occipital encephalocele, polymicrogyria, and heterotopia^(18)^. Most patients with Dandy-Walker malformation present with signs and symptoms of intracranial hypertension before one year of age^(18)^. Neuroimaging shows hypoplasia or, in rare cases, agenesis of the cerebellar vermis, which is elevated and upwardly rotated, together with cystic dilatation of the fourth ventricle^(18,19)^, as depicted in Figure 5. The cerebellar hemispheres are typically displaced anterolaterally, although with normal size and morphology. The posterior fossa is usually enlarged, and the tentorium is elevated^(18)^.

## DEGENERATIVE DISEASES

### Progressive ataxia and palatal tremor

**Figure 6:**
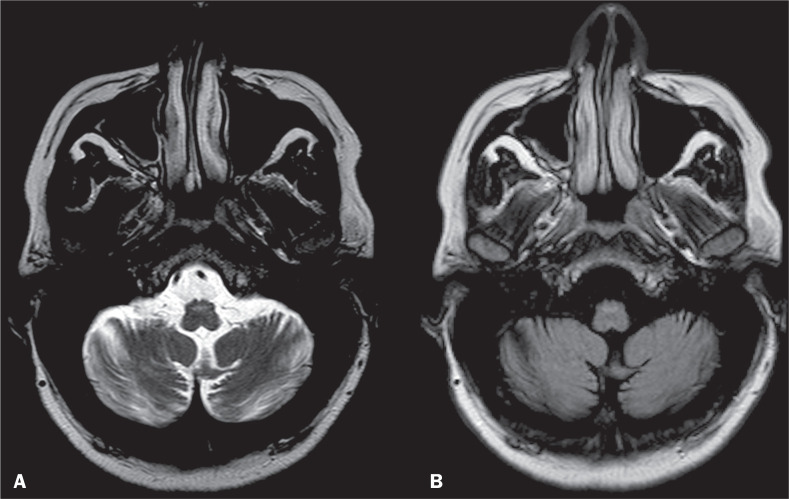
Marked brainstem atrophy, with a hyperintense signal on T2WI (A) and on a fluid-attenuated inversion recovery image (B) in the inferior olivary nuclei. The patient was diagnosed with PAPT.

Progressive ataxia and palatal tremor (PAPT) is a rare disorder which presents with palatal myoclonus and progressive cerebellar dysfunction^(20)^. It is most commonly a sporadic condition but may also be part of a familial disorder^(20)^. Clinical features of PAPT include visual disturbances, dysarthria, dysphagia, and arm ataxia^(20)^, as well as difficulty in walking and standing. When palatal tremor is accompanied by synchronous eye movements, it is known as oculopalatal tremor^(20)^. The imaging features of PAPT include hypertrophy and a hyperintense signal in the inferior olivary nuclei on T2WI and fluid-attenuated inversion recovery imaging, features that regress and can disappear in the chronic phases of disease. The disorder is also associated with cerebellar and brainstem atrophy (Figure 6). The main differential diagnosis of PAPT is hypertrophic olivary degeneration, in which the pathology of the palatal tremor is disruption of the Guillain-Mollaret triangle^(21,22)^.

## HEREDITARY DISEASES

### Friedreich’s ataxia

**Figure 7:**
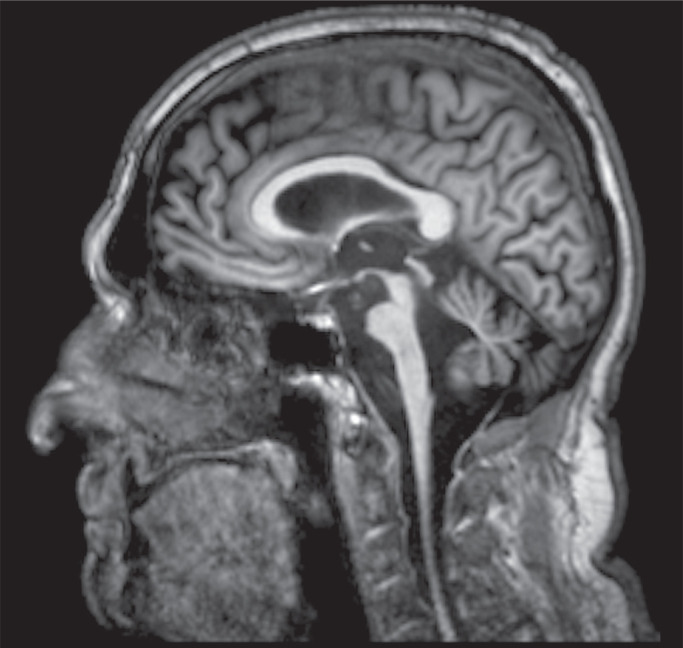
Sagittal T1WI showing marked atrophy of the cerebellum, pons, and spinal cord. The patient was diagnosed with Friedreich’s ataxia.

Friedreich’s ataxia is caused by the expansion of the GAA-triplet nucleotide sequence on chromosome 9q^(23)^. The length of the triplet repeat sequence determines the age at onset and the severity of the disease^(23,24)^. The GAA-triplet repeat is responsible for inhibiting transcription of the gene that encodes the mitochondrial protein frataxin, related to iron homeostasis^(23)^. Friedreich’s ataxia is an autosomal recessive multisystemic disorder that affects the central and peripheral nervous systems, the myocardium, the musculoskeletal system, and the endocrine pancreas^(24)^. The ataxia is caused by the combination of peripheral sensory neuropathy, spinocerebellar tract degeneration, and cerebellar pathology. The disorder typically appears before the age of 25 years, usually between 10 and 16 years of age, although cases of later onset have been reported^(24)^. The imaging features consist of atrophy of the cervical spinal cord, medulla, cerebellum, dentate nuclei, middle cerebellar peduncles, and pons (Figure 7). On T2WI, the signal in the lateral and posterior columns of the cervical spinal cord can be hyperintense^(23,25)^.

### Machado-Joseph disease

**Figure 8:**
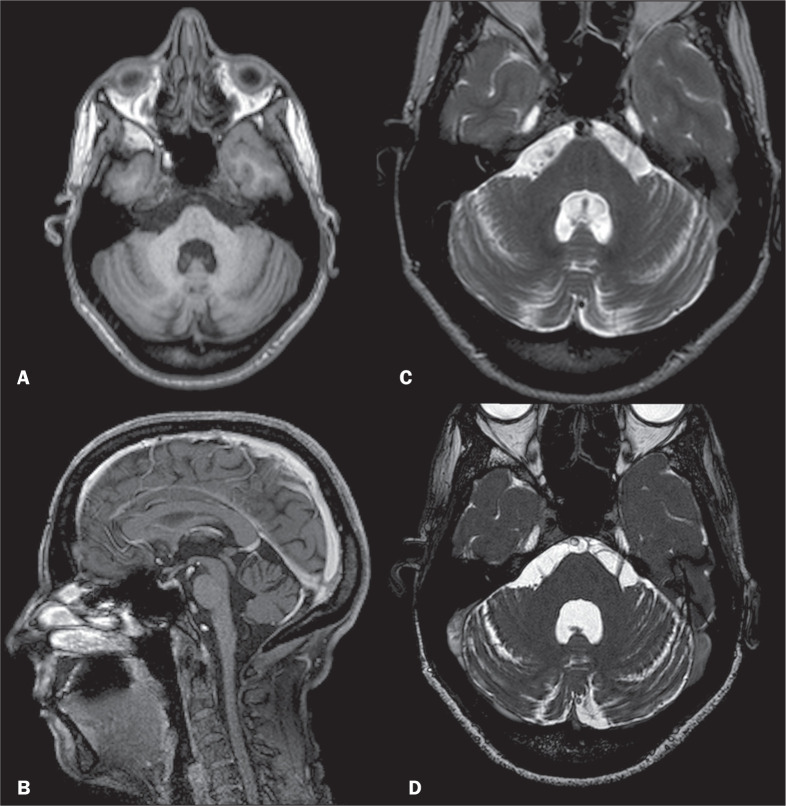
Axial T1WI (A), contrast-enhanced sagittal T1WI (B), T2WI (C), and axial fast imaging employing steady-state acquisition (D) showing atrophy of the middle cerebellar peduncles, enlarged cerebellar sulci, and generalized atrophy of the brainstem. The molecular diagnosis was Machado-Joseph disease.

Machado-Joseph disease, also known as spinocerebellar ataxia type 3, is a multisystem neurodegenerative disorder and the most common type of spinocerebellar ataxia^(26,27)^. The condition is caused by an unstable CAG repeat expansion at exon 10 of the ATXN3 gene, located on chromosome 14^(28)^. This mutation results in cerebellar degeneration^(27)^. Clinical findings include motor and non-motor manifestations, such as gait ataxia, ophthalmoplegia, hypokinetic/hyperkinetic disorders, parkinsonism, dystonia, myoclonus, chorea, dysautonomia, pain, cramps, fatigue, psychiatric disorders, olfactory dysfunction, peripheral neuropathy, and sleep disorders^(26,27)^. As shown in Figure 8, the MRI findings of Machado-Joseph disease include the following^(26,27)^: cerebellar and brainstem atrophy; frontal and temporal lobe atrophy; and marked atrophy of the superior cerebellar peduncle (characteristic of this condition), middle cerebellar peduncle, and globus pallidus.

## CONCLUSION

Ataxia is a syndrome that comprises multiple differential diagnoses and heterogeneous etiologies. As illustrated here, MRI is an important tool for determining the correct diagnosis.

## References

[1] Silva RN, Vallortigara J, Greenfield J (2019). Diagnosis and management of progressive ataxia in adults. Pract Neurol.

[2] AlRayahi J, Zapotocky M, Ramaswamy V (2018). Pediatric brain tumor genetics: what radiologists need to know. Radiographics.

[3] Alves CAPF, Fragoso DC, Gonçalves FG (2018). Cerebellar ataxia in children: a clinical and MRI approach to the differential diagnosis. Top Magn Reson Imaging.

[4] Klockgether T (2010). Sporadic ataxia with adult onset: classification and diagnostic criteria. Lancet Neurol.

[5] Klisch J, Juengling F, Spreer J (2001). Lhermitte-Duclos disease: assessment with MR imaging, positron emission tomography, single-photon emission CT, and MR spectroscopy. AJNR Am J Neuroradiol.

[6] Awwad EE, Levy E, Martin DS (1995). Atypical MR appearance of Lhermitte-Duclos disease with contrast enhancement. AJNR Am J Neuroradiol.

[7] Blumenthal GM, Dennis PA (2008). PTEN hamartoma tumor syndromes. Eur J Hum Genet.

[8] Fruehwald-Pallamar J, Puchner SB, Rossi A (2011). Magnetic resonance imaging spectrum of medulloblastoma. Neuroradiology.

[9] Eran A, Ozturk A, Aygun N (2010). Medulloblastoma: atypical CT and MRI findings in children. Pediatr Radiol.

[10] Mittal P (2011). Magnetic resonance spectroscopy findings in non-enhancing desmoplastic medulloblastoma. Ann Indian Acad Neurol.

[11] Yeom KW, Mobley BC, Lober RM (2013). Distinctive MRI features of pediatric medulloblastoma subtypes. AJR Am J Roentgenol.

[12] Beraldo GL, Brito ABC, Delamain MT (2019). Primary infratentorial diffuse large B-cell lymphoma: a challenging diagnosis in an immunocompetent patient. Rev Assoc Med Bras.

[13] Reis F, Schwingel R, Nascimento FBP (2013). Central nervous system lymphoma: iconographic essay. Radiol Bras.

[14] Koeller KK, Rushing EJ (2004). From the archives of the AFIP: pilocytic astrocytoma: radiologic-pathologic correlation. Radiographics.

[15] Aragao MFV, Law M, Almeida DB (2014). Comparison of perfusion, diffusion, and MR spectroscopy between low-grade enhancing pilocytic astrocytomas and high-grade astrocytomas. AJNR Am J Neuroradiol.

[16] Hanna MH, Bansal A, Belani P (2019). A review of radiographic imaging findings of ependymal tumors. Neurosurg Cases Rev.

[17] Yuh EL, Barkovich AJ, Gupta N (2009). Imaging of ependymomas: MRI and CT. Childs Nerv Syst.

[18] Bosemani T, Orman G, Boltshauser E (2015). Congenital abnormalities of the posterior fossa. Radiographics.

[19] Barkovich AJ, Kjos BO, Norman D (1989). Revised classification of posterior fossa cysts and cystlike malformations based on the results of multiplanar MR imaging. AJR Am J Roentgenol.

[20] Samuel M, Torun N, Tuite PJ (2004). Progressive ataxia and palatal tremor (PAPT): clinical and MRI assessment with review of palatal tremors. Brain.

[21] Gu CN, Carr CM, Kaufmann TJ (2015). MRI findings in nonlesional hypertrophic olivary degeneration. J Neuroimaging.

[22] Raeder MTL, Reis EP, Campos BM (2020). Transaxonal degenerations of cerebellar connections: the value of anatomical knowledge. Arq Neuropsiquiatr.

[23] De Michele G, Di Salle F, Filla A (1995). Magnetic resonance imaging in “typical” and “late onset” Friedreich’s disease and early onset cerebellar ataxia with retained tendon reflexes. Ital J Neurol Sci.

[24] Cook A, Giunti P (2017). Friedreich’s ataxia: clinical features, pathogenesis and management. Br Med Bull.

[25] Pagani E, Ginestroni A, Della Nave R (2010). Assessment of brain white matter fiber bundle atrophy in patients with Friedreich ataxia. Radiology.

[26] Murata Y, Yamaguchi S, Kawakami H (1998). Characteristic magnetic resonance imaging findings in Machado-Joseph disease. Arch Neurol.

[27] Kim Y, Kondo M, Sunami Y (2014). Stroke MRI findings in spinocerebellar ataxias. J Neurol Disord Stroke.

[28] Pedroso JL, França Jr MC, Braga-Neto P (2013). Nonmotor and extracerebellar features in Machado-Joseph disease: a review. Mov Disord.

